# The gastric microbiome, its interaction with *Helicobacter pylori*, and its potential role in the progression to stomach cancer

**DOI:** 10.1371/journal.ppat.1006573

**Published:** 2017-10-05

**Authors:** Jennifer M. Noto, Richard M. Peek

**Affiliations:** 1 Division of Gastroenterology, Department of Medicine, Vanderbilt University Medical Center, Nashville, Tennessee, United States of America; 2 Department of Pathology, Microbiology, and Immunology, Vanderbilt University Medical Center, Nashville, Tennessee, United States of America; 3 Department of Cancer Biology, Vanderbilt University Medical Center, Nashville, Tennessee, United States of America; Tufts Univ School of Medicine, UNITED STATES

## Introduction

**Fig 1:**
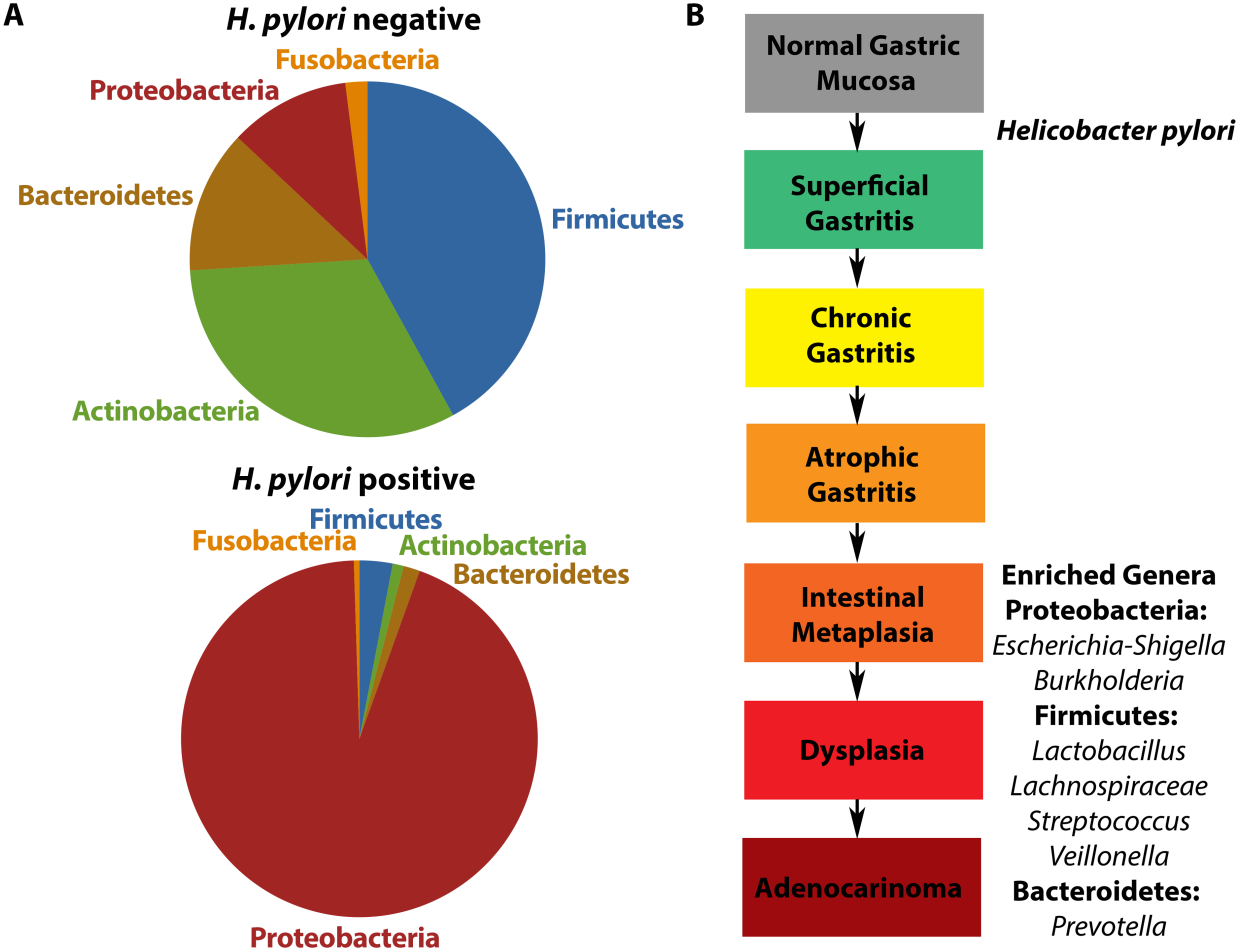
Alterations in the gastric microbiota following *Helicobacter pylori* infection and gastric disease progression. **(A)** Schematic representation of the predominant phyla of the gastric microbiota based on *H*. *pylori* infection status. *H*. *pylori*-negative individuals harbor a microbiota that is more complex and highly diverse compared to *H*. *pylori*-positive individuals. **(B)** Schematic representation of the predominant genera at different stages within the gastric carcinogenesis cascade. Following infection with *H*. *pylori*, Proteobacteria and specifically *H*. *pylori* dominate the gastric microbiota. This leads to the development of chronic gastritis. In the later stages of the disease, ranging from intestinal metaplasia to gastric adenocarcinoma, a number of genera are enriched. These include *Escherichia-Shigella* and *Burkholderia* within the Proteobacteria phylum; *Lactobacillus*, *Lachnospiraceae*, *Streptococcus*, and *Veillonella* within the Firmicutes phylum; and *Prevotella* within the Bacteroidetes phylum.

Gastric adenocarcinoma is the third leading cause of cancer-related death worldwide, accounting for more than 720,000 deaths annually [1]. The strongest known risk factor for this devastating disease is infection with *Helicobacter pylori*, which drives the development of premalignant lesions (such as gastric atrophy, intestinal metaplasia, and dysplasia) that can lead to gastric cancer (Fig 1). However, although *H*. *pylori* is the most common bacterial infection worldwide and colonizes greater than 50% of the global population, only 1%–3% of infected individuals ever develop gastric cancer.

Drivers of susceptibility to gastric carcinogenesis include *H*. *pylori* strain-specific virulence determinants, host constituents, and environmental factors. Along with these elements, the microbiota of the stomach may also influence the development of gastric malignancies. The acidic environment of the stomach in conjunction with low levels of cultured bacteria from this site previously led to assumptions that the gastric niche was not able to support a diverse microbial community. However, recent advances in DNA sequencing of conserved ribosomal RNA genes, phylogenetic analyses, and computational methods have uncovered a complex microbiota within the human stomach with the potential for disease induction [2].

## The gut microbiota and dysbiosis

The human gut microbiota is critical for maintenance of human health and plays an integral role in energy metabolism, absorption of nutrients, and defense against invading pathogens [3–5]. However, this microbiota exists within a delicate balance that, if altered, becomes dysbiotic and contributes to aberrant proinflammatory immune responses, susceptibility to invading pathogens, and initiation of disease processes, including cancer [6]. Dysbiosis contributes to the pathogenesis of gastrointestinal carcinomas in the esophagus [7] and colon [8, 9], and specific bacterial species are associated with the development of colorectal cancer (*Fusobacteria nucleatum* and *Escherichia coli*) [10, 11] and gastric cancer (*H*. *pylori*) [12].

The relationship between specific microbial pathogens and carcinogenesis has been the subject of extensive investigation, and historically, the majority of research has focused on individual pathogens, such as *H*. *pylori*, and their ability to initiate and perpetuate disease. Advances in sequencing technology have greatly enhanced the ability of scientists to identify additional microbial species that may be potentially associated with various disease states, such as cancer, although establishing cause versus effect presents multiple challenges. Therefore, understanding how dysbiosis impacts aberrant host inflammatory responses and downstream carcinogenic cascades will be critical to accurately define the role of niche-specific microbiota in oncogenesis.

## *H*. *pylori*, the human gastric microbiota, and gastric cancer

A first step in establishing causation is to take inventory of a particular resident microbial population, and several investigations have focused on defining microbial communities within the human stomach and the interactions of these populations with *H*. *pylori* (Fig 1A). Numerous groups have used PCR- and sequencing-based approaches to demonstrate that *H*. *pylori*-negative individuals harbor a highly diverse gastric microbiota dominated by 5 predominant phyla: Proteobacteria, Firmicutes, Actinobacteria, Bacteroidetes, and Fusobacteria [13–15]. In contrast, among *H*. *pylori*-positive subjects, *H*. *pylori* is the single most abundant bacterium present in the stomach and accounts for between 72% and 97% of all sequence reads [13, 14].

These initial studies primarily focused on defining the composition of the gastric microbiome stratified by *H*. *pylori* infection status but not disease diagnosis. Subsequent human cross-sectional studies have compared the gastric microbiota in patients with pathologic outcomes that span the gastric carcinogenesis cascade (Fig 1B). One study demonstrated that *H*. *pylori* was present at relatively low abundance in patients with advanced premalignant lesions and that the microbiota of patients with gastric cancer were dominated by species of *Lactobacillus*, *Streptococcus*, *Veillonella*, and *Prevotella* [16]. Another study demonstrated a steady decrease in bacterial diversity of the gastric microbiota, with an increasing abundance of *Lactobacillus* and *Lachnospiraceae* in patients progressing along the carcinogenic cascade [17]. The increase in these genera validates other studies that demonstrated similar increases in the abundances of *Lactobacilli* [16] and *Lachnospiraceae* [18, 19] in tissue samples from gastric cancer patients. The abundance of *Lactobacillus*, *Lachnospiraceae*, *Escherichia-Shigella*, *Nitrospirae*, and *Burkholderia* is also enriched when gastric cancer patients are compared to controls [19], supporting previous findings that *Lactobacillus* and *Lachnospiraceae* are present at higher abundance in gastric cancer [16–18, 20] and that *Escherichia-Shigella* is enriched in patients with colorectal cancer [21]. It is important to note that these studies only identified genetic evidence of bacteria and in-depth studies to assess viability of these organisms have not been performed. However, these results raise an intriguing hypothesis, namely that gastric colonization by non-*H*. *pylori* bacteria, many of which also colonize the intestine, could impact the risk for gastric cancer.

Most bacteria cannot survive in the acidic environment of the stomach. However, it has been well established that, in a subset of persons, infection with *H*. *pylori* leads to achlorhydria and decreased acid secretion. Thus, long-term *H*. *pylori* colonization and neutralization of the gastric environment may directly contribute to alterations in the gastric microbiota. There are also clinical studies that support this concept, namely that patients treated with acid-suppressive drugs, such as proton pump inhibitors, exhibit a significant increase in the burden of non-*H*. *pylori* bacteria within the stomach. Of interest, this increase correlates with increased inflammatory responses, suggesting that non-*H*. *pylori* bacteria that colonize an achlorhydric stomach may have the capacity to promote inflammation that could potentially facilitate the progression to cancer [22, 23]. However, definitive evidence for this requires careful interventional studies that have yet to be performed.

Although these studies demonstrate associations between the human gastric microbiota and *H*. *pylori* infection (as well as various *H*. *pylori*-induced pathologies), they do not directly differentiate cause from effect, primarily due to their cross-sectional study designs. One longitudinal study that supported the role of non-*H*. *pylori* species in the development of cancer assessed the effects of *H*. *pylori* eradication therapy on gastric cancer incidence over a 15-year time period [24]. Despite only a 47% eradication rate for *H*. *pylori*, there were similar reductions in the incidence of gastric cancer among subjects who received antibiotics and were unsuccessfully eradicated compared to those who remained *H*. *pylori*-free [24]. These results suggest that bacteria in addition to *H*. *pylori* may have been affected by antibiotics, which may have contributed to attenuated rates of gastric cancer. There are also computational biology studies that support these concepts. Using a computerized search algorithm designed to identify the presence of bacterial DNA within interrogated known cancer genomes, these investigators determined that the type of cancer that harbored the second highest number of bacterial DNA sequences was gastric adenocarcinoma. However, the most common type of integrated bacterial DNA was not *H*. *pylori* but was instead *Pseudomonas* [25].

## The effect of the gastric microbiota on *H*. *pylori*-induced gastric inflammation and cancer in rodents

The ability to establish causality is greatly enhanced by carefully controlled and manipulatable animal model systems. Inbred mice with defined genotypes are commonly used to study the effects of *H*. *pylori* infection on gastric diseases such as cancer. Of interest, the gastric microbiota of C57BL/6 mice is dominated by the same predominant phyla that have been reported in humans: Firmicutes, Bacteroidetes, Proteobacteria, and Actinobacteria [26]. Longitudinal studies in mice have provided more direct evidence of the contribution of non-*H*. *pylori* species to *H*. *pylori*-induced gastric carcinogenesis. For example, 1 study demonstrated that INS-GAS mice harboring a complex microbiota developed gastric cancer within 7 months following *H*. *pylori* infection, whereas the development of gastric cancer was markedly prolonged in germ-free mice that were monocolonized by *H*. *pylori* [27]. Following *H*. *pylori* infection, there was an overall decrease in gastric microbial diversity [27], similar to that observed in human populations, but there were no significant differences in the intestinal microbiota among any of the groups. These observations were studied in greater depth in an INS-GAS *H*. *pylori* mono-associated germ-free mouse model, where the addition of a restricted microflora accelerated the development of gastric cancer in conjunction with *H*. *pylori* [28]. Specifically, germ-free INS-GAS mice supplemented with a gastric and intestinal microbiota containing only 3 species of commensal intestinal bacteria (ASF356 *Clostridium* species, ASF361 *Lactobacillus murinus*, and ASF519 *Bacteroides* species) were sufficient to promote gastric neoplasia in *H*. *pylori*-infected INS-GAS mice to the same extent as observed in *H*. *pylori*-infected INS-GAS mice harboring a complex microbiota [28]. Importantly, these genera are also enriched in the stomachs of patients that develop premalignant and malignant lesions. Further supporting the concept of a contributory role of the gastric microbiota in promoting disease have been interventions with antibiotic therapy, which were shown to delay the onset of gastric cancer in INS-GAS mice in a manner that was not dependent on the presence of *H*. *pylori* [29]. Collectively, these results suggest that non-*H*. *pylori* bacteria can colonize the stomach and may represent an additional modifier of gastric cancer risk, particularly among *H*. *pylori*-infected individuals.

In addition to the stomach, bacteria within other microbial niches may exert a role in modulating *H*. *pylori*-induced gastric inflammatory responses. Two studies have shown that precolonization with intestinal *Helicobacters* (*H*. *bilis*, *H*. *hepaticus*, and *H*. *muridarum*) can either increase or decrease the severity of gastric inflammation induced by *H*. *pylori* by altering T-regulatory cell responses [30, 31]. Another study demonstrated that *H*. *pylori* per se is present within the intestine in a coccoid form and that the interaction between phagocytes and *H*. *pylori* within intestinal Peyer’s patches plays a critical role in modifying the intensity of *H*. *pylori*-induced gastritis [32]. However, other studies have shown that the microflora within the stomach can accelerate the progression of gastric cancer in the presence of *H*. *pylori* and do so with no differences detected in the composition of the intestinal microbiota [27, 28]. Addressing whether the resident intestinal microbiome directly contributes to the pathophysiology of *H*. *pylori*-induced gastric diseases is an avenue that requires further investigation, and it is important to consider that the effects of bacteria and microbial communities in the intestine and the stomach on gastric pathophysiology may not be mutually exclusive.

## Conclusions

Evidence that the host microbiota specifically functions to promote health and prevent disease and that dysbiosis contributes to inflammation, susceptibility to pathogens, and diseases (including cancer) is undisputed [3]. As a result, the concept of specific microorganisms solely driving cancer initiation and progression may need to be modified in certain circumstances. Although great advances have been made in understanding the complex interplay between the gastric microbiota and *H*. *pylori* in the development of gastric inflammation and cancer, detailed studies are still needed in well-defined human populations to compare differences in the microbiota of *H*. *pylori*-infected persons with and without neoplastic lesions. Cross-sectional studies can provide initial insights into microbial associations with cancer; however, reverse effects are a concern, as it is difficult to discern whether carcinogenesis leads to changes in the local microenvironment that creates a new niche for microbes or whether alterations in the microbial population or its functions contribute to carcinogenesis [33]. Due to the acidic nature of the stomach, most bacteria cannot survive in this environment. However, infection with *H*. *pylori* leads to achlorhydria of the stomach in a subset of colonized persons; thus, long-term *H*. *pylori* colonization and neutralization of the gastric environment may directly contribute to alterations in the gastric microbiota.

Since the gastric microbiota is more austere in terms of microbial breadth and depth compared to the intestinal microbiota, future studies should focus on assessing whether the composition of the gastric microbiome in different anatomical regions of the stomach exerts differential effects on cancer risk. This could be done through site-specific topographical mapping of the microbiota in the presence or absence of *H*. *pylori* as well as assessing differences in relation to different disease states along the gastric carcinogenesis cascade. Clearly, longitudinal studies that utilize sequential sampling to elucidate the temporal nature of microbial associations with premalignant lesions are needed. Details regarding patient populations, including age, gender, diet, and other comorbidities need to be assessed and compared in a rigorous fashion to discern whether any of these variables affect the potential for the gastric microbiome to influence disease. Since studies of the gastric microbiota have largely focused on bacterial communities, more in depth studies elucidating effects of other microorganisms that potentially populate the stomach in addition to bacteria, including fungi, protists, archaea, and viruses, are needed to fully characterize the gastric microbiome and its relationship with cancer risk. Furthermore, to more definitively determine cause versus effect, studies may also need to incorporate humanized mouse models to discern effects of the human gastric microbiome on disease. As we begin to understand and elucidate the specific role of the gastric microbiota and its effects on human health and disease, studies of these microbial populations in innovative systems will likely yield translational opportunities to reduce gastric cancer morbidity and mortality by improving screening, prevention, and treatment. It is tempting to speculate that future studies will identify specific combinatorial populations of bacteria that are predictive of pathologic outcomes, yielding strategies to manipulate the microbiota to ultimately prevent disease.
